# A qualitative study on relationships and perceptions between managers and clinicians and its effect on value-based healthcare within the national health service in the UK

**DOI:** 10.1177/09514848211068624

**Published:** 2022-02-08

**Authors:** Sze May Ng

**Affiliations:** 1 Department of Women’s and Children’s Health, 4591 University of Liverpool, Liverpool, UK; 2 7435 Southport and Ormskirk Hospitals NHS Trust, Ormskirk, UK

**Keywords:** value-based health, health care, clinical management, clinical managers

## Abstract

One of the main drivers for change towards delivering value-based healthcare is to improve clinical and managerial culture and engagement within organisations. The relationships between clinicians and managers in an organisation are often considered to be either an enabler or disabler towards successful engagement to develop strategies towards better value healthcare. Successful engagement is dependent on effective and transformational leadership that can impact on organisational value in healthcare. The aim of this research was to explore the relationships, behaviours and perceptions between managers and clinicians towards value-based healthcare in the National Health Service in the United Kingdom. A qualitative research methodology of semi-structured in-depth interviewing on a sample consisting of hospital consultants, senior managers and board executives from a diverse group were conducted. A thematic analysis was used to analyse the data using a systematic approach. The study findings identified areas of potential barriers to engagement for clinicians and managers which were related to regulatory burden, financial challenges and workforce shortages. Key recommendations on what will be required to improve clinicians and managers engagement and the leadership approaches towards improving value-based healthcare are discussed.

## Introduction

The National Health Service (NHS) has an increasing demand from the needs of its population and in times of austerity, there exist significant variations in care and health outcomes.^1,2^ The current responsibilities for leadership in the NHS must be shared across the system between clinicians and managers, while and an overly centralist approach will not work.^3^ Quality improvements and cost improvement strategies is not enough to meet the demand and challenges faced in the NHS today.^4^ The NHS Right Care Atlases of Variation demonstrates variations in resources within the UK, and highlights opportunities for improving the health service by delivering value-based outcomes.^5^ The Kings Fund also reported that one of the main drivers for change is managerial culture and engagement of clinicians and managers within organisations.^6^ Non-engagement between managers and clinicians has been a long-standing barrier towards quality improvements.^7^ Veronesi et al. reported that NHS organisations with larger proportions of clinicians in the executive board or leadership roles were more likely to achieve higher quality of care ratings and better staff and patient satisfaction.^8^ Managers can often find themselves fighting a losing battle to implement financially implicated change within an organisation without the engagement of clinicians. Porter describes that the objective of a successful healthcare system is to move away from a supply-driven healthcare delivery and to develop a person-centred system which revolves around what the patients need.^9^ The concept of delivering value-based health outcomes is a useful way to review healthcare delivery that is generated by cost and resources.^10^ The current shift from the traditional healthcare delivery model to a model based on value-based outcomes is inevitable. The emergence of new concepts around value-based healthcare will transform health services to deliver a triple value that will maximise value in healthcare and achieve the best value-based health outcomes for the population.^11^ Porter states that the first essential principle in creating a high-value healthcare delivery system is to define value as the goal while not reducing cost. Improving value requires improving outcomes per unit of cost. Managers of many organisations often pursue cost reduction as the key focus but in reducing costs without considering value, the organisation inadvertently end up leading to higher costs in the long run because they forgo smart investments or only postpone costs or move them to somebody else.^12^

### Current perceptions of clinical engagement

The NHS has invested significant amounts of time and money in leadership and organisation development to improve clinician-manager engagement which will lead to better value in healthcare, yet evidence of impact is variable. There may be perceptions that improving value is a euphemism for controlling and cutting cost rather than finding a better balance between delivering quality of care and the cost of delivering that care; however, change is needed to improve engagement of clinicians and managers to focus on delivering better value healthcare.^1^ There are considerable efforts to improve the value and quality of health in the NHS due to limited resources. Quality improvements have enormous potential to improve value-based outcomes, but it requires the buy-in jointly from both managers and clinicians.^13^ There is no universal definition of ‘clinical engagement’, and it may be defined as a perception, an attitude, a behaviour or an outcome.^12^ Such a collaborative partnership is often difficult to implement and sustain in the NHS, and the lack of clinical engagement in organisations have often been cited as the key to organisational failure. In this study, an exploration of what real engagement is and how it can be attributed to collaborative involvement is explored, as well as delving into the understating of what value in healthcare means to clinicians and managers are explored.

Board leadership have been strongly correlated with hospital performance and value-based health outcomes.^14^ Successful engagement between clinicians and managers will only take place if there is recognition at board level, and that this approach is cascaded across the directorates within an organisation.^14^ Clinical-managerial engagement and commitment to any change does not happen by chance, and it comes from senior leadership to nurture this engagement and a constant need to be checked, reinforced and worked upon.^2^ Many researchers have discussed if there was effective leadership from the board at the top level, this will translate to middle management and improve clinical engagement leading to improving organisational value in healthcare. Without more senior support, navigating cross-departmental obstacles, differences in perceptions and cultures between clinicians and managers become very challenging. It must be recognised that neither clinicians nor managers can make improvements or value-based progress in isolation as their collaboration is fundamental to sustainably embedding any quality improvements or innovations. The ever-increasing rate and pace of work, staffing crises, workforce issues can affect morale of clinicians and managers in the NHS. Board leadership must not ignore the need to take stock, support, reward, nurture, and value both clinical staff and managerial staff in their roles towards improving value in the health system.

There is much focus on the need to deliver high quality, value-based healthcare in the NHS and an emphasis on working in partnership between clinicians and managers to better engage and deliver. Despite significant attempts in recent years to increase clinical-managerial engagement in NHS, progress has been exceedingly slow.^14^ There remains a pressing need for research into how effective leadership can influence better engagement between clinicians and managers leading to better value-based outcomes, to understand drivers of engagement and to explore the behaviours and perceptions in order to make recommendations to improve their engagement and partnership working.

The aims of this study are to:

• explore the relationships, behaviours and perceptions between managers and clinicians towards value-based healthcare in the NHS.
• evaluate the relationships between the managers and clinicians when making decisions or implementing change.
• explore how leadership and engagement impacts on the organisation’s value in healthcare.

## Methodology

Studies and relevant papers related to the topics were identified using a standard search strategy which included searches from JSTOR, PROQUEST, SSCI, WILEY ONLINE, MEDLINE (1966 to November 2016) and EMBASE. A search strategy for MEDLINE was also used and accessed via UK government websites and PubMed based on the subject headings that were identified as keywords and terms of articles such as various combinations of keywords ‘Value’, ‘Value in health’, ‘Value based’ ‘Value based healthcare’, ‘clinicians’, ‘managers’, ‘NHS managers’, ‘NHS leaders’, ‘shared leadership’ and ‘distributed leadership’ crossed with Boolean connectors AND, OR or AND/OR.

A qualitative research methodology of semi-structured in-depth interviewing was used. The sample consisted of four hospital consultants, four senior managers and 4 board executives from a diverse group. The project was undertaken as part of the Healthcare Financial Management Association (HFMA) and BPP university MBA in healthcare business and finance consultancy project. The project was approved and reviewed by the research and ethics committee. Clinicians and managers were selected from a range of specialities recruited to ensure that there was a broad coverage of the main working areas within the NHS. The participants were also chosen from sites based on the review of their organisation’s performances and Care Quality Commission (CQC) ratings, such as NHS providers who were facing financial difficulties compared to those that were financially sustainable. These sites were selected because they allowed for evaluation of the cultures within the organisations as well as implementation factors across diverse patient populations and clinical settings.

Potential participants were identified between October 2019 and November 2019 and were sent an initial invitation. Written consent was obtained to participate in the interview. This was then followed up with a second email or telephone call to schedule the interview appointments. If ethical issues arose that could affect patient safety, then this would be raised with the relevant line managers and the organisation policy will be followed in these circumstances. Other potential ethical issues considered were evidence of misuse of power, bullying and harassment.

All interviews were transcribed and analysed with the digital recordings stored in a secured hard drive. Each participant’s understanding of the term value in healthcare, their organisation’s approach to value, the enablers, and barriers to implementing value improvement strategies were explored. Behaviours, engagement and culture between the managers and clinicians were also explored. A thematic analysis was used to analyse the data. Key themes such as meaning of value in healthcare, leadership, attitudes and perceptions between clinicians and managers in the organisations were reviewed. A systematic approach was further applied to analyse the themes extracted from the interview transcripts where it was important to compare and contrast data by themes, while also retaining the connection to other aspects of each individual’s account.

## Results

No ethical issues arose from the interviews conducted and the diversity of the group was noted. All participants gave verbal and written consent for the recorded interviews and consented to the recording of the interviews when the first email invitation was sent. All participants agreed to be acknowledged in this report. Three overriding themes were discussed and analysed concentrating on leadership, engagements and perceptions towards value in healthcare. The themes that arose from the analyses were related to a) perceptions of value in healthcare b) culture and behaviours towards organisation efficiencies c) teamwork and relationship building. There were also two further themes that emerged which were related to potential concerns with regard to e) trust and compliance and f) persistent workplace tensions in the NHS between clinicians and managers. (See narrative syntheses of results in Table 1).

**Table 1.** Thematic Analyses and summary of findings with exemplar quotes.

Themes	Summary findings and exemplar quotes
*Perceptions of value in healthcare*	Both clinicians and managers within their professions differed in their approaches to improving value in healthcare. Clinicians interviewed tended to focus value more on the patient aspect and often alluded to how the individual patient care was what mattered to the patient’s quality of life. They also expressed a sense of powerlessness where they are not in control of budgets and felt that they have little influence over the organisational goals which sat with the board. They expressed that the decisions they had to make would profoundly impact on the patients they see daily, the quality of care they deliver and their own reputation as doctors. In contrast, the executives and managers interviewed expressed a more generalised view of value in healthcare which focused on broad populations and wider allocation of resources within budgets in the organisation, with an aim to maximise efficiencies and value-based outcomes
	Participant quotes ‘Value in health outcomes is related to preventing admission, improving long term heath and improving patient experience’ (*manager*) ‘Value is the consideration of both cost and the quality of the service provided’ (*clinician)*
*Culture and behaviours towards organisation efficiencies*	Clinicians and managers observed important and similar aspects of integrating patient care and both parties understood the importance of improving value in health with the need to consider both cost and quality of the service. Clinicians and managers discussed similar aspects of maximising their organisational efficiencies with clear patient pathways. Their perceptions were similar in recognising a need for greater clinical input and leadership in implementing change and reforms. This would in turn enable the organisation to innovate and drive efficiency changes within hospital services
	Participant quotes ‘Value in healthcare is starting with the patient at the centre’ (*clinician)* ‘Getting the best possible outcome for every pound we spend in healthcare’ (*manager)*
*Teamwork and relationship building*	All participants mentioned the importance of being able to work together to articulate each other’s views when improving value-based outcomes. Managers expressed that many of them do not have all the skill sets to understand the clinical aspects, pathways or impact of an implementation. Conversely, clinicians do not have the managerial or financial knowledge that is required in such leadership roles
	Participant quotes ‘Getting the right clinical leaders and wrapping the right managerial and financial support around them are crucial’ (*manager)* ‘Both clinicians and managers really need each other to work together to make the right informed decisions’ *(clinician)*
*Persistent workplace tensions and burnout*	Clinicians and managers shared the same aims to improve value in health care, but their different perceptions can sometimes lead to tensions and conflicting approaches. Some of the workplace tensions encountered could be surmised by a quote ‘the managers are not on the frontline and often escape the wrath of the patients if care is compromised due to organisational budget cuts’ (clinician). Managers felt that workplace tensions exist in the NHS because their jobs were at stake, and therefore may feel pressured to go down a path just to achieve the cost improvement plans (CIPs), but may not see the safety and quality aspect of the implementation. There was consensus from both clinicians and managers interviewed that the NHS needs to break with the command and control, target-driven approach but to develop a shared leadership when implementing any change
	Participant quotes ‘Many clinicians at present do not have the time, feel burnout and undervalued- therefore any further roles asked of them in management becomes a greater burden when they are not supported- what doctors “can do” is not the same as what they “will do”’ (*clinician*) ‘If our managers impose a command and control, all it does is demoralises ground staff and clinicians and robs them of the authority to make decisions, tensions and poor care may follow’ *(clinician)* ‘Managers are often pressured into delivering CIPs, but clinicians know first-hand that any rash decision driven by short-term cost efficiencies can undermine patient quality, damage staff morale and cost more in the long term’ (*manager*)
*Trust and Compliance*	Mistrust was described among the clinicians when there was a perceived lack of transparency, consultation, or rational explanation for frequent change of priorities and implementation of cost improvement plans. This was a major source of frustration and often led to feelings of disengagement with managers and disempowerment, as well as a reduced appetite for clinical leadership. Managers felt that clinicians did not understand that the organisation relies on targets and aim to be sustainable and did not have a wider view of organisational aims
	Participant quotes ‘If clinicians fully understand the managers’ views, they are engaged. If they don’t fully understand, they don’t engage and become very suspicious. Managers need to show real life examples of how patient journey can improve the experience and the cost efficiency has to make sense’ *(clinician)* ‘Engagement needs to be seen through the lens of the person who is being engaged*’ (clinician)* ‘Clinicians have a fall back and does not “need any managerial role,” while managers fear losing their jobs if KPIs not achieved’ *(manager)* “In the end, doctors tend to listen to other doctors, that is far more powerful than using someone who is not a doctor if we want to influence change” (manager) ‘Clinicians and managers don’t know each other; therefore, they don’t trust each other. Managers are often concerned about the money spent and activity earned, but the clinician cares more about the patient he sees daily’ *(clinician)*

It was also noted that organisations that supported more distributed leadership were the ones that were achieving their financial targets and had outstanding CQC ratings, perhaps making it, therefore, easier for the organisation to adopt distributed leadership in that environment, rather than a command-control structure if they were to be a financially challenged organisation.

## Discussion

Qualitative methods in exploratory research uses open-ended questions and enabled the interviewer to probe while giving participants the opportunity to respond in their own words, as opposed to quantitative methods which has only fixed responses. The use of open-ended questions has the ability to evoke individual responses that are meaningful and culturally salient while being unanticipated in nature.^15^ Another advantage of qualitative methods is that they allow the flexibility for the interviewer to probe initial participant responses such as-why or how and so what questions.^15^

One of the most prominent themes from all the participants was the desire to improve value in the current NHS and there was a clear understanding for the need to create more value and quality for the same amount of money that was finite. Interestingly, the more successful and sustainable organisations were the ones where there was the greatest degree of alignment and engagement between clinicians and managers of their objectives, values and culture across the organisation. Clinicians from organisations that were rated as ‘excellent’ were more open and positive towards working in a collaborative relationship with managers while the clinicians from financially challenged trusts expressed greater issues with barriers and burnout.

It was evident from the study that there were differences in leadership approaches when examining the system-level and organisational level structures that may influence value in healthcare such as patient care, quality and cost of delivery leading to value in health care between the managers and clinicians. Strong clinical leadership and medical engagement at all levels was a feature of participants both clinicians and managers that came from higher performing organisations.^7,13^ It was also clear that successful organisational systems had developed cultures where their managers and clinicians were motivated and supported to work in partnership to optimise their different skills and expertise, experience and values to collaboratively achieve a high quality, productive and patient-focused value-based outcomes, for example, one of the organisations was innovative and successfully developed triumvirate leaderships consisting of clinician, manager and business finance analyst within their business units. Some organisations within the region were also in the process of developing business triumvirates that consist of clinician, manager and nurse leads which may perhaps lead to more focus on clinical health outcomes.

This research has identified areas of potential barriers to engagement for clinicians and managers which were related to regulatory burden, financial challenges, workforce shortages, risk of organisational failure, rapid staff turnover and there was a perception from the clinicians and managers interviewed, that these negative conditions seemed to be discouraging talented clinicians from coming forward to take senior leadership positions. There is currently no recognised pathway for working towards managerial roles and our medical career structures do not routinely expose doctors early in their career towards leadership or NHS management and operations. Clinicians are often sheltered from key areas in the organisation they work in, such as finance, performance and governance issues.^7^ Mistrust and workplace tensions has been shown in the study such that doctors believe that managers are more focussed on making decision that meet prescribed cost and performance targets rather than improve quality and clinical priorities.

Due to the limited time within the project timescales, a consideration would be to test the hypotheses with a larger audience by questionnaire using objective quantitative measures as well as qualitative methods in future larger studies. It is also possible that the group interviewed is not diverse or broad enough and therefore may be skewed towards medical clinicians or managers. Another limitation to the study was whether all clinicians or managers interviewed were truly motivated by the same things and do all they always know best?

The relevance of this research becomes clear on recognising the significant divide that has been identified between the cultures and perceptions of clinicians and managers in organisations that had not invested in developing closer working relationships or supporting leadership programmes. It has also been identified that often mistrust existed between clinicians and managers with both being dismissive of each other’s work, and that there was little connection between their contributions. Developing compassionate leadership requires acknowledging and making provision for the difficulties and challenges of working within the context of clinical and managerial roles.^17^

Disengagement between clinicians and managers within the NHS has been found to lead to mistrust and misunderstanding. This was noted in the study results. There is an imbalance between the deontology or ethics of duty of the doctor-patient relationship and the utilitarian nature of the NHS system that aims for the greatest good of the greatest number. At the basic level, the issues come down to the tensions within the NHS limited budgets to balance the system’s overall resources with demands on any one specific individual. Although managers have a natural utilitarian view, the clinician is faced with a deontological view of giving the best care to the individual patient in from of them. Doctors who are clinical managers are often pushed to adopt a utilitarian view of having to make the best use of the NHS^′^ finite resources. However, the relationships of doctor-patients are private and guarded by privacy and confidentiality and therefore intrinsically on a deontological approach. With two competing systems of deontology versus utilitarianism in operation within the NHS, rarely will managers and clinicians see eye to eye to reconcile, while neither fully appreciates nor may be aware of how powerful each viewpoint may be. Perhaps a way forward would be to reconcile clinicians and managers with an understanding of both strengths and weaknesses of both ethical views may improve the NHS service as a whole.

Key recommendations from this study on what will be required to improve clinician and managers engagement and their leadership approaches towards improving value-based healthcare in the NHS are shown on Table 2. Delivery of value-based healthcare in the NHS is still a relatively new concept. There appears to be minimal research that enquires into the leadership impact on value in healthcare and how this can affect organisational change. Value-based healthcare is a strategy that can provide a framework for identifying the need for improving quality of care and patients’ experiences within our healthcare service.^6^ Further research is needed to understand, develop and target rationalisation in order to deliver a value-based healthcare system for the NHS.

**Table 2.** Practical implications and recommendations from the study.

*Fostering teamwork, collaborations and removing any barriers to communications between clinicians and managers*		Organisations should aim to foster an open, honest, and transparent approach which include mutual respect, trust, visibility and close proximity. Developing close proximity working arrangements (e.g. sharing an office), allocated time for clinical managers in their roles and responsibilities will improve the clinician-manager relationships. Although doctors have reported on cynicism and mistrust regarding managerial motives, frequent informal interactions through proximity may help alleviate these uncertainties and build on trust and working relationships between professions^23^		
*Leadership training and opportunities*		Training and development programmes should be offered within the organisation to better engage and support clinicians and managers as a programme of work. An example suggested by a participant would be for managers to spend at least 10% of their time shadowing clinicians and remain in contact with them to learn their struggles. The same is true of clinicians interested in management roles, will need to spend at least 10% of their time learning about the latest financial and business trends		
*Formations of business unit triumvirates*		Facilitating working closely alongside managers and clinicians such as formations of business unit triumvirates with a clinician, finance manager and business analyst should be developed where clear links has been shown between an organisation’s performance and level of engagement between clinicians and managers. If such partnership working can become successful, morale in the organisation will rise and even difficult financial decisions can be supported and accepted with such distributed leadership approaches. Changes made to an organisation for efficiencies will invariably affect clinical practice which can directly affect clinicians’ work practices and therefore it is essential they understand why these changes are being made in the first place		
*Co-design quality improvements*		Any organisational quality improvements or innovations should be co-designed with clinicians, and clinicians’ participation should be supported. Managers and clinicians should jointly coordinate any improvement efforts with organisational goals, and both should be recognised and rewarded as part of a formal performance management process		
*Encouraging and talent-spotting more clinicians into leadership and management roles*		Developing a leadership programme and encouraging clinical leadership at every level- at board level, at divisional, at directorate level and at operational level. The clinical experience and knowledge of clinicians in managerial roles are crucial and distinct from the skills brought to the table by managers from a financial and business background with regards to improving value in healthcare. Recent clinical leadership initiatives include the development of the Medical Leadership Competency Framework.^24^ which moves away from training small numbers of top leaders to a model of shared and distributed leadership with a focus on supporting the development of leadership capabilities in all clinicians in all stages of their career		
*Formal leadership or management training*		Explore funded and complementary training for clinicians (for example, HFMA, NHS Leadership Academy) who wish to take on senior management roles such as qualifications and training of management skills and encourage doctors and some nurses to acquire management skills on the premise that professionals in frontline clinical practice are better placed to improve the operation of the NHS.^7^ Doctors who received leadership or management training generally perform better in leadership roles.^25^ The Academy of Medical Royal Colleges and the NHS Institute for innovation and improvement have developed management and leadership competency frameworks for all clinicians to develop and acquire appropriate management skills at key stages in their careers, moving away from training only small numbers of top clinical leaders late in their career to a model of shared and distributed leadership^15,24^		
*Shared leadership and engagement*		Organisations need to support shared leadership and engagement from exemplars at board level in delivering its value-based objectives, and to recognised clinician time needs to be ringfenced, for example, through effective appraisals, clear job design and a well-structured team environment. Organisations need to invest in formal leadership development, but also provide opportunities and avenues for future and developing leaders to hone their skills backed up by systems that support their leadership approaches		
*Distributed leadership*		Distributed leadership has been advocated in healthcare and is intended to engage and empower, so that power should be distributed more equally rather than in a form of ‘command and control’ within organisations. Staff at all levels should be empowered to make decisions and act upon them.^26^ A cohesive clinical-management group is therefore essential for improved value in health care		

There is growing evidence that in organisations where clinicians are more engaged in strategic planning and service improvements perform better than those where clinicians are alienated from the strategic plans of the hospital.^15^ As we continue to search for the holy grail of engagement and efficiency in the NHS, it is inevitable that the costs of running the NHS will always outstrip resources. The challenge in the NHS now is to use those available resources in an optimal way by engaging clinicians and managers into distributed leadership roles to improve value-based outcomes in an organisation. Board leadership should be open and transparent, while emphasising that business units and departments foster team working and collaboration while removing barriers to communication and innovation. Effective leadership should be shared and less reliant on single individuals but more on ownerships of teams. The study also showed that the more successful organisations were able to deliver a shift in culture and had better clinician-manager engagement, supported more leadership developments and allowed a diversified and shared leadership approach to flourish. Inevitably, the NHS today required managerial and clinical leaders who have learnt the skills of engagement and are able to collaboratively put them into practice as leaders.

Although researching this project, what was striking was not the differences between the clinicians and managers but rather some stark similarities in their struggles and the need to be able to understand each other to provide a collaborative and distributed leadership. Although vertical systems were previously effective, it was clear that in the current NHS climate of budget cuts, targets and aims to increase value-based outcomes, hierarchical systems become more of an obstacle as change and strategy is required.^18^ The study findings have improved the gaps in the existing literature.

In undertaking this research, another similarity noted was that both clinicians and managers understood the importance and concepts of adding value in healthcare. Both had similar perceptions, for example, that adopting Lean and Kaizen methodologies in their organisations will add value-based outcomes and provide better quality of care with less waste of resources, through application of continuous quality improvements.^19^ There is growing evidence that the NHS cannot be led by professional managers alone, but it is increasingly important to integrate clinicians into shared leadership roles. This is essential for delivering good value-based health and quality outcomes.^20^ New conceptions of leadership are needed, and these will demand new leadership approaches. Systems leadership describes a collaborative network of people from different backgrounds, cultures and different levels in the system working towards a shared vision to enable a significant change’ resonates with me during this project.^21^ Systems leadership is almost the opposite of the ‘command and control’ approach and has elements of transformational leadership approaches^22^ which focuses on relationship building, ability to understand change and sharing knowledge. There were more similarities rather than differences in perceptions, cultures and behaviours between ‘clinicians’ and ‘managers’, and this research has certainly dispelled some of the pre-existing myths of divisiveness between clinicians and managers within their NHS leadership roles. A new model of distributed clinician-managerial leadership should be further explored where there is equal partnership between the clinical profession and those with business and financial experience.

Engagement in healthcare is far too important to be left to chance, and it needs individual organisation’s explicit strategy for both clinicians and managers to work together to give the biggest returns on resources used. Further research into what truly influences clinician-manager engagement as they work together towards meeting the demands of improving value in healthcare would be valuable.

Limitations of the study’s use of qualitative approach were that while it allowed flexibility as it consisted of open-ended questions, the structure and process may still be influenced by the characteristics of the researcher therefore critical reflection throughout the research process is paramount.^16^ Often, qualitative studies may also not be a true representation of the population, therefore may not be able to distinguish differences as well as quantitative research can when applied. In some cases, respondents may provide inaccurate information – or say what they think the researcher wants to hear.^16^

## Summary of new findings

• Successful organisations had better clinician-manager engagement and supported more clinical leadership developments,
• A diversified and shared leadership approach should be encouraged,
• Managerial and clinical leaders who have learnt the skills of engagement and are able to collaboratively work together better as leaders in the NHS.
